# Genetic Polymorphisms in *SCN1A* Gene (rs6432860) and Pharmacoresistance to Antiepileptic Drugs Among Jordanian Patients with Epilepsy

**DOI:** 10.3390/ph19050712

**Published:** 2026-04-30

**Authors:** Hanen Al-Sadir, Ayat Al-Farhood, Al-Motassem Yousef, Rami Abduljabbar, Shayma Abdullah, Ali Abuhaliema, Violet Kasabri

**Affiliations:** 1 Department of Biopharmaceutics and Clinical Pharmacy, School of Pharmacy, University of Jordan, Amman 11942, Jordan; h93snowwhite@yahoo.com (H.A.-S.); ayat93.hk@yahoo.com (A.A.-F.); ramiabduljabbar@gmail.com (R.A.); s.abdullah@ju.edu.jo (S.A.); 2 Clinical Pharmacy and Therapeutics in Pharmacological and Diagnostic Research Center, Faculty of Pharmacy, Al-Ahliyya Amman University, Amman 19111, Jordan; a.abuhaliema@ammanu.edu.jo

**Keywords:** epilepsy, antiepileptic drugs, *SCN1A*, seizure, drug resistance, single-nucleotide polymorphisms

## Abstract

**Background:** We investigated whether common variants in *SCN1A* are associated with antiepileptic drug (AED) non-response in Jordanian patients with epilepsy. **Methods:** We recruited 114 patients (105 successfully genotyped) and Sanger-sequenced five loci spanning rs6432860 and its flanking region of rs1531380, rs1531379, rs1531378, and rs10198801. Genotype–response associations were tested using contingency analyses and multivariable logistic regression adjusting for age at the time of the interview, number of AEDs, and carbamazepine use. Pre-specified secondary analyses included (i) stratification by AED class (voltage-gated sodium channel [VGSC]-acting vs. non-VGSC agents) and (ii) sensitivity analyses using alternative non-response thresholds (seizures > 0/year and ≥4/year). Linkage disequilibrium (LD) and exact Hardy–Weinberg equilibrium (HWE) tests were evaluated. Cohort minor allele frequencies (MAFs) were compared with global population estimates. **Results:** The four upstream previously cataloged intronic variant SNPs (rs1531380, rs1531379, rs1531378, and rs6432860) were in a complete pattern of LD association in this population (D′ = 1; r^2 =^ 1) whereas each upstream variant with rs10198801 showed D′ = 1 with inverse correlation (r ≈ −0.53). All loci conformed to the exact HWE tests. Upstream variants had novel associations with a non-response in unadjusted analyses and remained significant after adjustment (genotype aOR = 2.8; 95% CI = 1.1–7.2; *p* value = 0.03), alongside independent effects of carbamazepine use (aOR = 3.3; 95% CI = 1.3–8.0; *p* value = 0.009) and a number of AEDs (aOR = 0.17; 95% CI = 0.06–0.50; *p* value = 0.002). In AED-class stratification, upstream additional intronic variants had novel associations with a non-response among VGSC-treated patients (OR = 3.8; 95% CI = 1.1–13.6; *p* value = 0.03) whereas rs10198801 was associated among non-VGSC patients (OR = 7.9; 95% CI = 0.9–70; *p* value *=* 0.04). Findings were robust using a ≥4 seizures/year threshold (recessive model significant) but not using any seizures > 0/year. Cohort MAFs for upstream variants (~48.6%) exceeded European, African, and Asian estimates. **Significance:** SCN1A upstream intronic variation has a novel association with AED non-response in the Jordanian cohort, shows mechanism-aligned patterns by AED class, persists after covariate adjustment and under a clinically used seizure-frequency threshold, and warrants ancestry-informed replication and functional validation.

## 1. Introduction

Epilepsy is a neurological disorder characterized by a persistent tendency to experience epileptic seizures [1,2]. As one of the most prevalent neurological disorders, epilepsy affects over 50 million people globally, impacting individuals of all ages, with the highest risks observed in infants and older adults [3]. In approximately half of the individuals with epilepsy, the cause is not identifiable. However, various factors can contribute to seizure development, including brain trauma, high fever or severe illness, stroke (accounting for 50% of epilepsy cases in older adults), brain tumors, cerebral hypoxia, mental conditions such as Alzheimer’s disease, infections like AIDS, HIV, or meningitis, maternal medication use during pregnancy, prenatal injuries, brain malformations, birth asphyxia, and genetic, developmental, or neurological disorders [4]. Antiepileptic drugs (AEDs) are the primary treatment approach for epilepsy, though 20–30% of patients experience seizures that are not controlled by medication (drug-resistant epilepsy, or DRE) [4,5]. Managing DRE, particularly in children, poses significant challenges, leading to economic, social, and personal burdens, including reduced quality of life, cognitive impairments, and mood disruptions [6].

### 1.1. Operational Definitions of Drug-Resistant Epilepsy (DRE)

International guidelines from the International League Against Epilepsy (ILAE) define drug-resistant epilepsy (*DRE*) as the failure of adequate trials of two tolerated, appropriately chosen, and appropriately used antiseizure medication (ASM) regimens, whether as monotherapies or in combination, to achieve sustained seizure freedom. Sustained seizure freedom is generally operationalized as ≥12 months seizure-free or ≥3× the longest pre-intervention inter-seizure interval, whichever is longer [7]. This consensus was established to standardize reporting and facilitate earlier specialist referral [8].

Notwithstanding this consensus, epidemiologic and pharmacogenetic studies of alternative thresholds in sensitivity analyses have sometimes adopted pragmatic seizure-frequency thresholds to dichotomize responses, for example “>1 seizure/year” or “≥4 seizures/year”, particularly when long, prospectively adjudicated remission data are unavailable. Such choices can materially alter the apparent prevalence of DRE and the magnitude of genotype–phenotype associations. Recent adult cohorts comparing concurrent definitions have shown that the proportion classified as DRE varies meaningfully across criteria, underscoring the need to state and justify the operational definition used [9].

DRE is thought to result from a combination of genetic and non-genetic factors, and leveraging an individual’s genetic profile to predict the drug response and efficacy is a key goal [3,5,10,11,12,13,14]. Many AEDs achieve anticonvulsant effects by blocking voltage-gated sodium channels (VGSCs), which play an essential role in generating and transmitting action potentials in neurons and other excitable cells [15]. VGSCs are vital for initiating action potentials and facilitating their transmission throughout the nervous system, making them central to both the development and mitigation of epilepsy [15,16,17]. Genetic factors significantly influence epilepsy’s phenotypic expression, presenting challenges due to variable expression within families and substantial genetic heterogeneity. Recent advances, such as genetic hybridization, have enabled the identification of additional genomic regions and genes associated with various epilepsy disorders and related pathophysiological mechanisms [17]. Several SNPs have been linked to AED dosage requirements and drug resistance [18,19,20,21,22,23,24].

The *SCN1A* gene, also known as the sodium channel alpha-1 subunit gene, is an ion channel with a potential role in epilepsy’s pathophysiology. The selected SNP for this study was chosen due to its reported prevalence (>5%), and existing research suggesting a relevant association. *SCN1A* genes are among the genes that encode for the alpha subunit of VGSCs. SCN1A encodes for the alpha “pore-forming’’ subunit of just one of nine known sodium channels. The protein produced by SCN1A is commonly known as Nav1 1. It is in chromosome 2, consists of 29 exons, and spans 164,521 nucleotides (NC_000002.12: 165,984,641 to location 166,149,161 on chromosome 2) on the complement side on the long arm q24.3. It is five times over-expressed in brain tissue [25]. Findings from this study could enable clinicians to customize treatment based on patients’ genetic profiles, thereby improving clinical outcomes and advancing our understanding of SNPs within the *SCN1A* gene [26].

### 1.2. Aims

Given this landscape, we pre-specify our primary DRE definition and acknowledge the heterogeneity in the literature. To enhance clinical interpretability and generalizability, we also conducted sensitivity analyses using frequency-based thresholds aligned with prior work (e.g., any breakthrough seizures >0/year and ≥4/year) and compared the results across thresholds. The aim of this study was to investigate the potential association of genetic polymorphisms in the *SCN1A* gene (rs6432860) with resistance to AEDs in Jordanian epilepsy patients.

## 2. Results

### 2.1. Subject’s Demographic Descriptions and Clinical Characteristics

A total of 114 epilepsy patients participated in this study. According to their drug response, patients were divided into two groups: 65 (57%) were responsive to AEDs, while 49 (43%) were not. Age categories were defined as tertiles of the cohort distribution to form three approximately equal groups: <22 years, 22–39 years, >39 years (statistical, not clinical cut-off points). Clinical characteristics among responders and non-responders are summarized in Table 1. Responders to AEDs were younger at the time of interview (27.9 ± 12.9 vs. 36.2 ± 12.1 year; *p* value = 0.001) and younger at the onset of epilepsy diagnosis (18.4 ± 12 vs. 25.1 ± 12.8 year; *p* value < 0.01). Responders received less of the AED, that is, fewer AEDs (mean number of concomitant AEDs 1.5 ± 0.7 vs. 2 ± 0.8 year; *p* value = 0.001) were delineated. The non-responders were more likely to be treated with valproate (OR = 3.2; 95% CI = 1.4–7.2; *p* value < 0.01) or carbamazepine (OR = 3.9; 95% CI = 1.8–8.5; *p* value = 0.001; Table 1).

### 2.2. Association of Alleles and Genotype Polymorphisms of SCN1A Gene with Response to AEDs

All genotypes of the studied SNPs were consistent with exact Hardy–Weinberg equilibrium tests across the entire sample (N = 105) as well as within both responders and non-responders (*p* value > 0.05, Chi-Square, df = 1; Table 2). Of the 32 possible haplotypes, three were identified in the following SNP order: rs1531380, rs1531379, rs1531378, rs6432860, and rs10198801. The most common haplotype across the entire sample was TAAAG (48.6%), followed by CGGGG (28.6%) and CGGGT (22.9%), while TAAAT was not observed (0%). The TAAAG haplotype was more frequent among non-responders (57% vs. 42.7%; *p* value = 0.042), whereas the CGGGG haplotype (25.6% vs. 30.7%; *p* value = 0.4) and the CGGGT haplotype (17.4% vs. 26.6%; *p* value = 0.12) were more common among responders, although these differences were not statistically significant. There was strong and statistically significant linkage disequilibrium (LD) between any two of the four upstream SNPs (D = 0.25; D′ = 1; r = 1; *p* value < 0.001) and also between the fifth SNP and each of the four upstream SNPs (D = −0.11; D′ = 1; r = −0.53; *p* value < 0.001). Pairwise LD (D, r^2^) is shown in Figure S1 LD (Supplementary), with the underlying numeric values in Table S3 LD (Supplementary).

A summary of genotype and allele frequencies of the five SNPs is presented in Table 2 as it enlists the *SCN1A* gene’s genotypes and alleles. All four upstream SNPs were associated with a response to AEDs while the fifth SNP (rs10198801) was not. Carriers of the homozygous recessive allele genotype were four times more likely to be non-responders (OR = 4; 95% CI = 1.1–14.5; *p* value = 0.03) when compared to the homozygous dominant allele genotype, for all four upstream SNPs. The recessive allele was 1.8 times more likely to be found among the non-responders for all four upstream SNPs (OR = 1.8; 95% CI = 1.02–3.1; *p* value = 0.042). Moreover, exact HWE test *p* values (overall and by response subgroup) are provided in Table S1 HWE (Supplementary); no deviations from exact HWE tests were detected (Supplementary).

Because of the imbalance in the distribution of some baseline variables (age, number of AED, and the use of valproate or carbamazepine) among responders and non-responders, backward stepwise logistic regression was utilized to predict the best set of variables to predict the lack of response to AEDs. The logistic regression was specified for a significance level of 0.05 for entry and at a level of 0.1 for removal. The final analysis revealed that genetic polymorphism at rs6432860 retained a statistically significant association in response to AED (a OR = 2.8; 95% CI = 1.1–7.2; *p* value = 0.03).

### 2.3. AED-Stratified Analysis by Voltage-Gated Sodium Channel (VGSC) Blockade

Given the mechanistic heterogeneity of AEDs used by participants (Table 1), we conducted a stratified analysis according to exposure to VGSC-acting AEDs (carbamazepine, lamotrigine, and topiramate) versus non-VGSC AEDs (e.g., valproate, levetiracetam, and gabapentin). In the subgroup limited to patients receiving VGSC blockers, genetic polymorphisms in the four upstream intronic SNPs (rs1531380, rs1531379, rs1531378, and rs6432860) were significantly associated in response to AEDs while the downstream SNP (rs10198801) was not. Carriers of at least one recessive allele genotype at any upstream SNP were 3.8 times more likely to be non-responders compared with carriers of the homozygous dominant genotype (OR = 3.8; 95% CI = 1.1–13.6; *p* value = 0.03). Conversely, in the subgroup limited to patients not receiving any VGSC blocker, only the downstream SNP (rs10198801) was significantly associated in response, whereas the four upstream SNPs were not. In this subgroup, carriers of the homozygous dominant allele genotype were 7.9 times more likely to be non-responders when compared with carriers of at least one recessive allele genotype (OR = 7.9; 95% CI = 0.9–70; *p* value = 0.04). While statistically significant, the wide confidence interval indicates imprecision consistent with a smaller subgroup size and warrants cautious interpretation. These findings suggest differential genetic associations by pharmacologic subclass, with upstream intronic variants associating with resistance among VGSC-treated patients, and rs10198801 associating with resistance among non-VGSC-treated patients (see Table 1 for AED distribution; 71 of 114 patients received at least one VGSC-acting AED).

### 2.4. Sensitivity Analysis by Seizure-Frequency Threshold

Principally because definitions of treatment response vary in the literature, we re-classified outcomes using two alternative thresholds: any breakthrough seizures ≥ 1/year and ≥4/year and re-tested SNP-response associations (Table 1 for AED distribution).

(a) Any breakthrough seizures

Using this cut-off as the non-response threshold, no statistically significant genotype–outcome associations were observed across tested models. This likely reflects the conservative nature of the threshold (targeting absolute seizure freedom), potential titration phases, and smaller effective group contrasts (29 responders vs. 76 DRE).

(b) Seizures ≥ 4/year

Under the ≥4/year threshold, the upstream SNP showed a borderline association (Pearson χ^2^ = 5.495, df = 2, *p* value = 0.064) when the three genotypes were considered. The recessive model (TT vs. CC + CT) showed significant results (OR = 3.2, 95% CI = 1.1–9.4, *p* value = 0.04). TT was more frequent among patients with seizures ≥ 4/year (77.3%) than 0–3/year (22.7%), consistent with a recessive-model risk effect.

### 2.5. Multivariable Logistic Regression Adjusting for Clinical Covariates

To account for imbalances in baseline characteristics (age at interview, number of AEDs, and carbamazepine use), we fit backward stepwise logistic regression models with the treatment response status (non-response vs. response) as the dependent variable and each SNP entered under a dominant genetic model (minor-allele carriers vs. major-allele homozygotes), alongside covariates. Across all four upstream SNP models (rs1531380, rs1531379, rs1531378, and rs6432860), the genotype term remained statistically significant, with identical adjusted odds ratios (aOR = 2.8; 95% CI = 1.1–7.2; *p* value = 0.03). In the same models, carbamazepine use showed an independent association with non-response (aOR = 3.3; 95% CI = 1.3–8.0; *p* value = 0.009), number of AEDs (aOR = 0.17; 95% CI = 0.06–0.50; *p* value = 0.002), and age at interview (aOR = 0.30; 95% CI = 0.10–0.61; *p* value = 0.002; Table 3).

## 3. Discussion

Epilepsy is a common episodic neurological disorder with a wide range of clinical manifestations. Genetics and its variations are a novel hypothesis believed to be the primary factor underlying the condition in approximately 70% of individuals with epilepsy who do not have an identifiable external etiology. Most epilepsy phenotypes result from the interaction between genetic and environmental factors [27]. VGSCs play a critical role in membrane excitability and action potential generation. They are heteromeric complexes that regulate sodium exchange between intracellular and extracellular. The genes encoding these channels consist of one α subunit and two β subunits, making them key targets for improving AED efficacy. Different genes encode the isoforms of these channels. Allelic variants of this gene are associated with generalized epilepsy, febrile seizures, and epileptic encephalopathy [18,20,21,22,23,24,28]. Previous studies have suggested that genetic polymorphisms in genes encoding VGSCs are involved in the lack of response to AEDs [18,20,21,22,23,24].

The purpose of our investigation was to determine the relationship between AED resistance and various SNPs of the *SCN1A* gene (rs1531380, rs1531379, rs1531378, rs6432860, and rs10198801) among Jordanian patients with epilepsy. Our study included 114 epileptic individuals, categorized as either drug-responsive (N = 65) or drug-non-responsive (N = 49).

The prevalence of AED responsiveness is significantly influenced by the definition of AED resistance used, which varies considerably across studies. A comprehensive list of these definitions was published in a 2014 meta-analysis [29]. Sunwoo et al. [30] defined drug resistance as experiencing more than one seizure per year, identifying 74% of participants as having drug-resistant epilepsy (DRE) [30,31]. In contrast, Denton et al. [9] classified resistant patients as those having more than four seizures annually, identifying 59% of current participants as DRE. For our study, we adopted the definition from Haerian et al. [19], which categorizes approximately 50% of patients as non-responsive; our findings indicated a non-responsive percentage of 43% among the participants [19]. The overall prevalence of non-responsiveness to antiepileptic drugs (AEDs) was significantly higher than previously reported [29]. The observed non-responsive rate of 43% in our cohort is higher than the ~20–35% often cited in population-based estimates of drug-resistant epilepsy (DRE). Several factors likely contribute. First, there is the case mix and setting. The sampling was recruited from a single center, a high-throughput public hospital that serves lower socioeconomic communities (Al Bashir). In this setting, patients face greater clinical complexity, delayed access, or barriers to continuous care (selection/referral bias). Second, operational definitions matter. We show in our sensitivity analyses that the prevalence varies when applying stringent seizure freedom criteria versus pragmatic frequency thresholds, consistent with other adult cohorts when comparing concurrent definitions. Third, treatment heterogeneity/polytherapy, differential adherence, and unmeasured syndrome-level variation may elevate the apparent non-response in real-world cross-sections. Together, these contextual factors help explain the higher point estimate while reinforcing the need for larger, multicenter cohorts using harmonized ILAE aligned endpoints. This noteworthy finding should be validated with a larger sample size that includes patients from medical facilities beyond Al-Bashir Hospital.

The rs6432860 SNP was selected due to the availability of published literature and its reported minor allele frequency (MAF) of >5%. We decided to sequence the flanking regions upstream and downstream of this SNP to identify previously cataloged intronic SNPs and explore their association with AED response. This study identified four previously cataloged intronic SNPs (rs1531380, rs1531379, rs1531378, and rs10198801) in addition to rs6432860. The first three were located upstream, and the last was downstream. Interestingly, the three upstream SNPs were in complete linkage with each other and rs6432860 (D’ = 1, r^2^ = 1, *p* value < 0.001), resulting in identical counts and percentages for all genotypes and alleles. This distinct pattern was consistent across the entire sample, as well as among responders and non-responders. The physical distance between these SNPs ranged from 10 to 648 bp, potentially explaining the completeness (LD). A distinctly strong LD was also observed between rs6432860 and the downstream SNP, rs10198801 (D’ = 1, r = −0.53, *p* value < 0.001).

The MAF of the four upstream SNPs was 48.6% among Jordanian patients with epilepsy, which is notably higher than the frequencies in apparently healthy individuals from Asia (13%), Africa (19%), and Europe (33%) [3,5,10,11,12,13,14], all of which differ substantially from our findings. Further investigation is warranted to confirm this result; however, these findings suggest that the SNPs may contribute to a heightened susceptibility to epilepsy.

**Figure 1:**
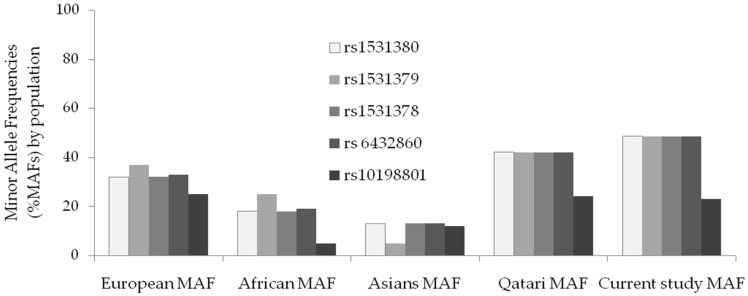
Published minor allele frequencies (MAFs) by global populations vs. the current Jordanian cohort.

A literature search failed to reveal any publications related to the previously cataloged intronic SNPs (rs1531380, rs1531379, rs1531378 or rs10198801), all of which were in the flanking region of rs6432860 [32,33]. To our knowledge, no prior reports have evaluated associations with AED non-response for rs1531380, rs1531379, rs1531378, or rs10198801 in a Jordanian cohort; thus, our observation represents a population-specific association rather than the discovery of new variants. This aligns with both new MAFs in Table 4 and Figure 1, which demonstrate these SNPs as being present globally and quantify how the Jordanian cohort differs and supports the “not novel variants, but population-specific profile/association” framing. On the other hand, there were six publications related to rs6432860; the oldest was published in 2013 and the most recent in 2020. Two publications ascertained the role of rs6432860 in the development of febrile seizures [9,29,34,35,36,37,38].

### 3.1. Racial Differences or Variations in Genotype and Allele Frequencies

Conflicting results across studies likely arise from differences in phenotype definition (including DRE criteria), ancestry and MAF structure, inclusion/exclusion criteria, and environmental or health system context, in addition to study size and statistical correction strategies. Such heterogeneity is well described in DRE research and underscores the importance of transparent definitions and replication in diverse, prospectively enrolled cohorts. A meta-analysis by Li et al. [39] observed a wide range of minor allele frequencies (MAFs) across ethnic groups [39,40,41]. Inconsistent data reported in these epidemiological studies may also result from factors such as differences in inclusion and exclusion criteria, isolated study populations, epilepsy heterogeneity, and gene–environment interactions, among other unidentified variables. A key contributor to these discrepancies is the variation in definitions of “AED resistance” used across studies [39,41,42]. A large Australian study (N = 519) did not find any association between rs6432860 and responsiveness to AEDs [39]. The lack of a statistically significant association in the Australian cohort (approx. N = 519) likely reflects phenotype and design heterogeneity rather than a contradiction of our findings. Specifically, that study evaluated the SCN1A variation in the context of febrile seizures (including vaccine-related febrile seizures) rather than ASM responsiveness in epilepsy, i.e., a different outcome construct with distinct age distribution and pathophysiology. Differences in case definition, endpoint adjudication, ancestry composition, medication exposure, and multiple testing corrections further attenuate the power to detect modest pharmacogenetic effects across studies. Our discussion and sensitivity analyses therefore frame the present association as context-dependent and hypothesis-generating, warranting prospective replication under harmonized DRE criteria.

Meanwhile, an Indian study (N = 478) revealed that carriers of the AC haplotype (A allele from rs6432860 and C allele from rs3812718) were 2.7 times more likely to suffer from recurrent seizures in patients treated with phenytoin (N = 94) [32]. This finding parallels the results from the current study, where the A allele was associated with more of a tendency towards a lack of response to AEDs (OR = 1.8; 95% CI = 1.02–3.09; *p* value = 0.042).

To contextualize potential racial differences and gene–environment interactions [43,44,45], we compared minor allele frequencies (MAFs) in our cohort with published population estimates. For the four upstream variants, our cohort exhibited higher MAFs (~48.6%) than those reported in Europe (32–37%), Africa (18–25%), and Asia (5–13%), and was comparable to or higher than Qatari estimates (~42%). In contrast, the downstream variant rs10198801 in our cohort (22.9%) was similar to Asian (12%) and European (25%) ranges but higher than African ones (5%). This pattern illustrates meaningful population stratification and helps explain the variability in association signals across studies, emphasizing the need for replication in diverse cohorts and careful adjustment for ancestry (Table 4; Figure 1).

### 3.2. AED Mechanism Heterogeneity and Subgroup-Specific Associations and Their Mechanistic Plausibility

Notably, 71 of 114 patients in our cohort were treated with at least one AED with established or partial VGSC-blocking activity (carbamazepine, lamotrigine, or topiramate) while others (e.g., valproate, levetiracetam, and gabapentin) act predominantly through alternative mechanisms (Table 1). In stratified analyses, the four upstream intronic variants (rs1531380, rs1531379, rs1531378, and rs6432860) were significantly associated with drug resistance among patients receiving VGSC-acting therapy whereas the downstream variant rs10198801 was associated with resistance among patients not receiving VGSC blockers. The upstream signal in the VGSC subgroup is biologically congruent with the locus under study and supports the plausibility that regulatory or splicing-related intronic variation may influence sodium-channel function and consequently the pharmacodynamic effects of VGSC-acting AEDs. In contrast, the rs10198801 novel association in the non-VGSC subgroup may reflect an alternative regulatory element or a distinct marker of linkage disequilibrium (LD) with loci that modulate non-VGSC pathways. The subgroup-specific patterns, along with the persistence of significance under stratification, collectively argue against a single-drug artifact and instead suggest a mechanism-aligned genetic susceptibility. Nonetheless, the wide confidence interval observed in the non-VGSC subgroup underscores the need for larger, prospectively enrolled cohorts and functional studies to delineate causal mechanisms and to determine whether these SNPs exert direct effects or serve as proxies for nearby functional variation.

An additional consideration is the heterogeneity of antiepileptic drug (AED) mechanisms in our cohort. Although Table 1 shows that patients received multiple AED classes, the majority (71/105; 67.6%) were treated with at least one agent with established or partial voltage-gated sodium channel (VGSC)-blocking activity (carbamazepine, lamotrigine, or topiramate) while others (e.g., valproate, levetiracetam, and gabapentin) act predominantly through alternative pathways. This pharmacologic profile is congruent with the biology of the locus under study and may help explain the strength of the observed association between *SCN1A* variants and drug resistance; variants that influence sodium channel function or its regulation could be expected to have a greater clinical impact when VGSC-acting therapies are in use. At the same time, the persistence of a statistically significant association for rs6432860 in our multivariable model indicates that the signal is unlikely to be solely attributable to a single drug class and may also reflect broader effects on neuronal excitability (e.g., regulatory or splicing consequences of intronic variants) or linkage disequilibrium with other functional loci. Given our sample size and co-medication patterns, we were underpowered for a fully stratified analysis by individual AED; we therefore highlight, as a priority for future work, stratified or class-specific analyses (and gene–drug interaction testing) in larger cohorts to determine whether the association is primarily driven by VGSC-acting agents or is consistent across pharmacologic subclasses.

Moreover, when data were limited to only those with VGSC blockers (carbamazepine, lamotrigine, or topiramate), there was a statistically significant association between DRE and genetic polymorphism. All four upstream SNPs were associated in response to AEDs while the fifth SNP (rs10198801) was not. Carriers of at least one recessive allele genotype were 3.8 times more likely to be non-responders (OR = 3.8; 95% CI = 1.1–13.6; *p* value = 0.03) when compared to a homozygous dominant allele genotype, for all four upstream SNPs. Furthermore, when data were limited to only those not taking any of the VGSC blockers (carbamazepine, lamotrigine, or topiramate), there was a statistically significant association between DRE and the genetic polymorphism. Remarkably, only the downstream SNP (rs10198801) had novel association in response to AEDs while all the four upstream SNPs did not. Carriers of a homozygous dominant allele genotype were 7.9 times more likely to be non-responders (OR = 7.9; 95% CI = 0.9–70; *p* value = 0.04) when compared to carriers of one recessive allele genotype.

### 3.3. Sensitivity Analysis by Seizure-Frequency Threshold

Essentially, the definition of drug-resistant epilepsy varies across studies and guidelines. Our primary classification used twelve seizures/year to distinguish non-responders, consistent with the approach adopted from Haerian et al. [19]; however, other reports in our discussion have employed >1/year and ≥4/year thresholds (e.g., Sunwoo and Denton), which are closer to routine clinical practice. To address this concern, we performed sensitivity analyses using alternative thresholds: any breakthrough seizures >1/year and ≥4/year. The >1/year threshold did not produce significant genotype–response associations, likely reflecting an ultra-stringent clinical target and titration periods that blur group contrasts. In contrast, the ≥4/year threshold yielded significant associations consistent with a recessive genetic model (e.g., TT vs. CT + CC, MH OR = 3.2, 95% CI 1.17–9.4, *p* value = 0.04), reinforcing the biological plausibility of our findings under a widely used clinical threshold. Together with our AED-class stratification and multivariable modeling, these results suggest that the observed associations are not artifacts of a single classification scheme, but remain detectable under clinically relevant thresholds while still warranting confirmation in larger, prospective cohorts with standardized definitions.

### 3.4. Limitations

This study has several limitations that should be considered when interpreting the findings.

1. Sample size and statistical power

Although 114 patients were recruited, only 105 samples were successfully genotyped (9 were excluded due to DNA extraction/PCR/sequencing issues), which constrains power particularly for subgroup and interaction analyses (e.g., by AED class or individual drugs). The findings of this study should be interpreted carefully within the context of its limitations. The small sample size and the single-center location (Al-Basheer Hospital) may limit the generalizability of the results. However, similar studies have recruited samples that are comparable in size or even smaller than ours. In addition, we did not apply multiplicity corrections (e.g., Bonferroni), which increases the risk of type I error for secondary tests. These factors call for cautious interpretation and validation in larger cohorts.

2. Single-center, hospital-based sampling

The overall prevalence of non-responsiveness to AEDs was substantially higher than previously reported [29]. Moreover, participants with a high rate of resistance were recruited exclusively from Al-Bashir Hospital over a relatively short period (January 2021–November 2022). As Al-Bashir primarily serves patients from lower socioeconomic backgrounds, selection bias cannot be excluded, and the results may not generalize to other clinical settings or populations. Financial burden remains a significantly critical unmet medical need, particularly in cases of refractory epilepsy [30,31,32,33,34,35,36,37,38,39,40,41]. Multicenter cohort recruitment across diverse care settings would improve external validity.

3. Outcome classification heterogeneity

Definitions of drug-resistant epilepsy (DRE) vary across the literature, and our primary operational definition differs from other commonly cited thresholds. Variability in seizure-frequency cut-off points across studies (e.g., >1/year or ≥4/year) raises the possibility of outcome misclassification that could attenuate or inflate genotype–response associations. Harmonized, guideline-aligned endpoints are needed in future prospective work.

4. Confounding and measurement bias

Although multivariable models adjusted for the age at the interview, number of AEDs, and carbamazepine use, unmeasured or imperfectly measured confounders may persist (e.g., epilepsy syndrome, disease duration, EEG/imaging correlates, and comorbidity burden). Medication adherence was assessed using the Morisky Medication Adherence Scale (MMAS), which, while validated, relies on self-report and remains subject to information bias.

5. Medication exposure heterogeneity and polytherapy

Patients received multiple AED classes and often combination therapy, and certain drugs were more common among non-responders (e.g., carbamazepine and valproate). While we presented class-stratified analyses, small cell sizes and treatment heterogeneity limit the precision and the ability to ascribe associations to specific agents without indication bias. Larger datasets will be required for stable, drug-specific inference.

6. Genetic scope and functional inference

The work targeted a 1193 bp region around rs6432860 with five SNPs genotyped by PCR and Sanger sequencing. This focused approach does not capture broader genome-wide variations, structural variants, or distal regulatory elements. Only a limited number of SNPs were explored in this study, despite the large number of SNPs in the SCN1A gene. Although the SCN1A gene has many SNPs (N = 29,377), only a few are cited in published articles and have a minor allele frequency (MAF) > 5% (n = 47). Among these, one is a missense variant (rs2298771, MAF = 21%), and two are synonymous variants (rs6432860, MAF = 21%; rs7580482, MAF = 29%), while the remaining variants are intronic (n = 44) [36,42,46]. This study focused specifically on rs6432860 and the nearby flanking region. Moreover, the associated intronic variants lack functional validation, precluding causal inference from gene expression, splicing, or protein function. Future studies should include functional assays and expanded genomic coverage.

7. Population specificity and stratification

The cohort reflects a single Jordanian population. As we show in our MAF comparison, the upstream variants have higher minor allele frequencies in this cohort than published estimates for European, African, and Asian populations while rs10198801 is closer to global ranges. This emphasizes both the importance of population-specific replication and the need to account for ancestry in genetic association studies.

8. Attrition/missingness

The exclusion of nine samples due to laboratory failures may introduce attrition bias if missingness is not completely at random; although unavoidable, this further underscores the need for larger, prospective cohorts with robust biospecimen QC procedures.

### 3.5. Future Research Directions

Collectively, these limitations lead us to argue for a larger prospective study in individuals with newly diagnosed epilepsy from various medical facilities across different socioeconomic levels, adequately powered studies with harmonized DRE definitions, richer clinical phenotyping, ancestry adjustment, expanded genomic coverage, functional validation of intronic variants and the role of the previously cataloged intronic SNPs to determine causality and therapeutic relevance and to validate AED pharmacokinetics and pharmacodynamics.

### 3.6. Current Perspectives, Clinical Significance, and Implications

From a clinical perspective, our findings suggest that these SNPs could potentially serve as biomarkers for predicting the AED response, contributing to personalized medicine in epilepsy. The *SCN1A* gene and its associated pathways may also represent potential therapeutic targets for novel AEDs. However, further studies are required to validate these findings and translate them into clinical practice.

## 4. Materials and Methods

Epileptic patients were recruited in accordance with the Declaration of Helsinki [47] from the Neurology Clinics at Al-Bashir Hospital between November 2021 and January 2022. During this period, both samples and relevant information were collected. Each participant was interviewed, and a comprehensive data collection form was completed. Collected data included the patient age, gender, seizure frequency, personal and family clinical history, lifestyle details, date of initiation of antiepileptic medication therapy, and other pertinent information.

Patients were eligible to participate if they were diagnosed with epilepsy by a neurologist and had been on AED therapy for at least one year. Exclusion criteria included a diagnosis of genetic abnormalities related to epilepsy, refusal to provide informed consent, and non-compliance with AED therapy, as assessed by the Morisky Medication Adherence Scale-8 (MMAS-8) [48]. Non-adherence was defined as MMAS-8 < 8(low adherence and medium adherence), and such patients were excluded, in line with prior validation studies [48]. For the outcome definition, we adopted the “≥12 seizures over one year” threshold to (i) harmonize with prior pharmacogenetic work that dichotomized the AED response using frequency-based operational criteria in cross-sectional designs, and (ii) ensure conservative separation between responders and non-responders in a hospital-based cohort in which adjudicated, long-term remission endpoints were not uniformly available. We explicitly recognize the ILAE consensus definition of drug-resistant epilepsy (failure of two appropriately chosen and used ASMs to achieve sustained seizure freedom) and that different operationalizations can alter the observed prevalence and effect sizes. Accordingly, we performed sensitivity analyses using any breakthrough seizures >0/year and ≥4 seizures/year thresholds (as used in prior studies) to assess the robustness and clinical generalizability of the genetic associations.

### 4.1. Sample Size Calculation

The sample size for responders and non-responders was calculated using Cochran’s formula N = (PQZ^2^)/D^2^ [49]. In this formula, N represents the sample size, Z is 3.24 (1.96 + 1.28, accounting for a 5% two-sided α and a 10% β), P is the estimated frequency (21%) of the MAF (A) allele for rs6432860 [50] and D is the meaningful difference (20%)(MAF) between responders and non-responders. The calculated sample size was 43 per group. However, to account for potential sample loss, incomplete patient data, non-cooperation, and reaction failures (DNA extraction, sequencing, and PCR), we increased the sample size by 25%, resulting in a target sample size of 114.

### 4.2. Ethical Considerations

This study was conducted with full respect to participant confidentiality and rights, under approvals from Al-Bashir Hospital, the Jordanian Ministry of Health, the Deanship of Academic Research, and the Institutional Review Board (MOH/REC/2021/57). Written informed consent was obtained from each patient, ensuring that data would be used solely for research and confidentiality would be maintained, and participants retained the right to withdraw from this study at any time.

### 4.3. Genotyping and Sequencing

A Promega-Wizard genomic DNA purification kit was used to extract the genomic DNA from 105 blood samples obtained from epileptic patients who routinely visit the Neurology Outpatient Clinic at Al-Bashir Hospital. Initially, 114 blood samples were targeted for collection. However, 9 samples were excluded due to challenges encountered during DNA extraction, PCR amplification, and sequencing, resulting in a final sample size of 105. Exact HWE tests were assessed using exact two-sided tests on observed genotype counts. In addition, LD metrics (D′, r^2^) and haplotype frequencies were estimated in Haploview v4.2 (EM algorithm).

The *SCN1A* gene’s SNPs rs1531380, rs1531379, rs1531378, rs6432860, and rs10198801 were amplified by a polymerase chain reaction (PCR), followed by sequencing using the Sanger Dideoxy Method. Appropriate forward and reverse primers were designed in order to amplify *SCN1A* (1193 bp) (Table 5). All variants have rsIDs and are previously cataloged. In this study, their frequency, LD structure, and associations in a Jordanian epilepsy cohort were recorded. The PCR conditions were as follows: 10 μL of One Taq^®^R Quick-Load VR master mix, 1.5 μL (10 μM) of each forward and reverse primer, 4 μL of nuclease-free water, and 2 μL (50–100 ng) of genomic DNA. Then, thermal cycler programs were performed using master mix protocols and various optimizations, such as first denaturation for 30 s at 94 °C, then another cycle of denaturation for 30 s at 94 °C, annealing for 5 min at 68 °C, extension for 60 s at 68 °C, and a last extension for 5 min at 68 °C. The agarose gel was then stained with RedSafe^TM^ (ChemBio Ltd., Porters Wood, St. Albans, UK, AL3 6PA) to enable the visualization of the PCR products. PCR purification and Sanger sequencing were performed by GENEWIZ Technical Support Group, USA (http://www.genewiz.com). In sequencing quality control and replication, PCR amplicons were subjected to bidirectional Sanger sequencing, where each sample was sequenced once with the forward primer (plus strand) and once with the reverse primer (minus strand) to confirm all variant calls on both strands. In addition, a 15% random subset of samples was re-sequenced in independent runs as an internal reproducibility check. Discrepant or low-quality base calls were flagged from trace inspection and re-read (and re-sequenced if necessary) before final genotype assignment.

### 4.4. Genotypes, Alleles, and Haplotype Determinations

Alleles and genotype frequencies of the five SNPs were estimated from PCR-sequencing data [50]. The interaction between genetic polymorphisms at the five loci was assessed by haplotype analysis and linkage disequilibrium (LD) [51,52,53]. LD was performed using Haploview software (Version 4.2). LD was quantified by calculating the LD coefficient (D), standardized D (D′), correlation coefficient (r), and *p* value [54].

### 4.5. Data Management and Statistical Analysis

The Statistical Package for Social Sciences (SPSS) version 22 (SPSS ^®^ Inc, Chicago, LA, USA) program was used to analyze the data. Quantitative data were summarized as the average ± standard deviation while qualitative data were summarized as counts and percentages. Genotype and allele frequencies for different alleles among the patients under study were calculated using the Chromas Lite software version 2.1.1’s sequencing and reading results. Chi-square (df = 1) was used to assess genotype and allele frequencies in accordance with the exact Hardy–Weinberg equilibrium tests. The Mantel Hanzel common odds ratios (ORs) and the 95 percent confidence intervals (CIs) were computed to assess the correlation between the categorical variables. The choice of univariant analysis was made in light of the data’s normality and homogeneity of variance.

Hypothesis testing of qualitative data was conducted utilizing chi-square or the Fisher’s exact test as appropriate, while the ANOVA independent *t*-test, Kruskal–Wallis test, or Mann–Whitney test was used to compare quantitative data. Multivariant analysis was carried out using backward stepwise logistic regression. No attempts were made to apply the Bonferroni correction of α. To determine the degree of association, the adjusted odds ratio (OR) was employed. Statistical significance was determined by a *p* value of less than 0.05.

## 5. Conclusions

Genetic polymorphisms of the SCN1A gene (rs1531380, rs1531379, rs1531378, and rs6432860) were fully concordant, and their minor alleles were linked to resistance to AEDs, while rs10198801 was not associated with resistance to AEDs. The association was observed at the level of genotypes, alleles and haplotypes.

## Figures and Tables

**Table 1:** Comparison of clinical and patient characteristics according to response to AEDs.

Variables	Categories	Total Patients (N = 114)		Non-Responsive Patients (N = 49)		Responsive Patients (N = 65)		* OR	95% CI	*p* Value
		N	%	N	%	N	%			
Gender	Male	61	53.5	31	63.3	30	46.2	3.3	1.5–7.5	0.1
	Female	53	46.5	18	36.7	35	53.8			
Age at interview (years)	<22 years	34	30	6	12.2	28	43.1	0.18	0.07–0.49	<0.01
	22–39 years	45	39.4	24	49	21	32.3	2	0.94–4.3	0.07
	>39 years	35	30.7	19	38.8	16	24.6	1.9	0.87–4	0.1
Age at seizure onset (years)	<14 years	37	32.5	11	22.4	26	40	1	(Reference Category)	
	14–25 years	43	37.7	18	36.7	25	38.5	1.7	(0.67–4.3)	0.26
	>25 years	34	30	20	40.8	14	21.5	3.4	(1.3–9)	0.014
Family history	Yes	17	14.9	5	10.2	12	18.5	0.5	0.16–1.5	0.22
	No	97	85	44	89.8	53	81.5			
Number of AEDs	Monotherapy	53	46.5	14	28.6	39	60	1	(Reference Category)	
	Bitherapy	43	37.7	22	44.9	21	32.3	2.9	(1.2–6.9)	0.013
	≥3 AEDs	18	15.8	13	26.5	5	7.7	7.2	(2.2–24)	0.0005
AEDs prescribed	Valproate	69	60.5	37	75.5	32	49.2	3.2	1.4–7.2	0.0045
	Carbamazepine	51	44.7	31	63.3	20	30.8	3.9	1.8–8.5	0.001
	Levetiracetam	41	36	18	36.7	23	35.4	1.1	0.5–2.3	0.88
	Lamotrigine	21	18.4	10	20.4	11	16.4	1.3	0.5–3.3	0.81
	Topiramate	14	12.3	5	10.2	9	13.8	0.7	0.22–2.3	0.58
	Gabapentin	3	2.6	1	21	2	3.11	0.66	0.058–7.5	0.99

Notes: For values expressed as N and %, N is number of patients and % was calculated out of available data. AEDs: antiepileptic drugs. Fisher’s exact test or chi-square tests (as appropriate) were used to estimate *p* value; *p* value < 0.05 is considered significant. OR: odds ratio. CI: confidence interval. Odds ratio and its 95% CI were calculated for *p* value> 0.05. *p* value was calculated for the three groups together using chi-square test. * OR was calculated in reference to non-responders. (i) Age grouping: tertiles were <22, 22–39, or >39 years; (ii) Tests: Pearson’s chi-square or Fisher’s exact test, as appropriate; *p*-values for multi-level variables are for overall comparisons; (iii) Odds ratios (ORs): model the odds of non-response; OR > 1 indicates higher odds of non-response; for multi-level variables, ORs are relative to the level marked “Reference category;” (iv) Abbreviations: ASM is antiseizure medication; OR is odds ratio; CI is confidence interval.

**Table 2:** Genotype and alleles frequencies of *SCN1A* polymorphisms.

SNP	Genotype	Total Patients (N = 105)		Non-Responsive Patients (N = 43)		Responsive Patients (N = 62)		OR	95%CI	*p* Value
		N	%	N	%	N	%			
rs1531380 166040707	CC	25	23.8	5	11.6	20	32.3	(Reference Category)		
	CT	58	55.2	27	62.8	31	50.0	3.5	(1.15–10.5)	0.023
	TT	22	21.0	11	25.6	11	17.7	4	(1.1–14.5)	0.03
	CT + CC	83	79.0	32	74.4	51	82.3	(Reference Category)		
	TT	22	21.0	11	25.6	11	17.7	1.6	(0.62–4.1)	0.33
	TT + CT	80	76.2	38	88.4	42	67.7	(Reference Category)		
	CC	25	23.8	5	11.6	20	32.3	0.28	(0.094–0.81)	0.015
	C allele	108	51.4	37	43.0	71	57.3	(Reference Category)		
	*T* allele	102	48.6	49	57.0	53	42.7	1.8	(1.02–3.09)	0.042
rs1531379 166040716	GG	25	23.8	5	11.6	20	32.3	(Reference Category)		
	AG	58	55.2	27	62.8	31	50.0	3.5	(1.15–10.5)	0.023
	AA	22	21.0	11	25.6	11	17.7	4	(1.1–14.5)	0.03
	AG + GG	83	79.0	32	74.4	51	82.3	(Reference Category)		
	AA	22	21.0	11	25.6	11	17.7	1.6	(0.62–4.1)	0.33
	AA + AG	80	76.2	38	88.4	42	67.7	(Reference Category)		
	GG	25	23.8	5	11.6	20	32.3	0.28	(0.094–0.81)	0.015
	G allele	108	51.4	37	43.0	71	57.3	(Reference Category)		
	A allele	102	48.6	49	57.0	53	42.7	1.8	(1.0–3.09)	0.042
rs1531378 166040738	GG	25	23.8	5	11.6	20	32.3	(Reference Category)		
	GA	58	55.2	27	62.8	31	50.0	3.5	(1.15–10.5)	0.023
	AA	22	21.0	11	25.6	11	17.7	4	(1.1–14.5)	0.03
	GA + GG	83	79.0	32	74.4	51	82.3	(Reference Category)		
	AA	22	21.0	11	25.6	11	17.7	1.6	(0.62–4.1)	0.33
	AA + GA	80	76.2	38	88.4	42	67.7	(Reference Category)		
	GG	25	23.8	5	11.6	20	32.3	0.28	(0.094–0.81)	0.015
	G allele	108	51.4	37	43.0	71	57.3	(Reference Category)		
	A allele	102	48.6	49	57.0	53	42.7	1.8	(1.0–3.09)	0.042
rs6432860 166041354	GG	25	23.8	5	11.6	20	32.3	(Reference Category)		
	GA	58	55.2	27	62.8	31	50.0	3.5	(1.15–10.5)	0.023
	AA	22	21.0	11	25.6	11	17.7	4	(1.1–14.5)	0.03
	GA + GG	83	79.0	32	74.4	51	82.3	(Reference Category)		
	AA	22	21.0	11	25.6	11	17.7	1.6	(0.62–4.1)	0.33
	AA + GA	80	76.2	38	88.4	42	67.7	(Reference Category)		
	GG	25	23.8	5	11.6	20	32.3	0.28	(0.094–0.81)	0.015
	G allele	108	51.4	37	43.0	71	57.3	(Reference Category)		
	A allele	102	48.6	49	57.0	53	42.7	1.8	(1.02–3.09)	0.042
rs10198801 166041507	GG	62	59.0	29	67.4	33	53.2	(Reference Category)		
	GT	38	36.2	13	30.2	25	40.3	0.59	(0.26–1.36)	0.22
	TT	5	4.8	1	2.3	4	6.5	0.28	(0.03–2.69)	0.37
	GT + GG	100	95.2	42	97.7	58	93.5	(Reference Category)		
	TT	5	4.8	1	2.3	4	6.5	0.35	(0.037–3.2)	0.65
	TT + GT	43	41.0	14	32.6	29	46.8	(Reference Category)		
	GG	62	59.0	29	67.4	33	53.2	1.8	(0.81–4.1)	0.15
	G allele	162	77.1	71	82.5	91	73.4	(Reference Category)		
	T allele	48	22.9	15	17.4	33	26.6	0.58	(0.29–1.2)	0.12

Fisher or chi-square test (as appropriate) were used to estimate *p* value; *p* value of <0.05 is considered significant. For values expressed as N and %, N is the number of patients and % was calculated out of the above data.

**Table 3:** Multivariable logistic regression adjusting for clinical covariates.

Covariate/Genetic Term	Model: rs1531380 (TT + CT vs. CC)	Model: rs1531379 (AA + AG vs. GG)	Model: rs1531378 (AA + GA vs. GG)	Model: rs6432860 (AA + GA vs. GG)
Carbamazepine (Yes vs. No)	aOR 3.3 (1.3–8.0); *p* value = 0.009	aOR 3.3 (1.3–8.0); *p* value = 0.009	aOR 3.3 (1.3–8.0); *p* value = 0.009	aOR 3.3 (1.3–8.0); *p* value = 0.009
Number of AEDs	aOR 0.17 (0.06–0.50); *p* value = 0.002	aOR 0.17 (0.06–0.50); *p* value = 0.002	aOR 0.17 (0.06–0.50); *p* value = 0.002	aOR 0.17 (0.06–0.50); *p* value = 0.002
Age at interview (years)	aOR 0.30 (0.10–0.61); *p* value = 0.002	aOR 0.30 (0.10–0.61); *p* value = 0.002	aOR 0.30 (0.10–0.61); *p* value = 0.002	aOR 0.30 (0.10–0.61); *p* value = 0.002
Genotype (dominant model)	aOR 2.8 (1.1–7.2); *p* value = 0.03	aOR 2.8 (1.1–7.2); *p* value = 0.03	aOR 2.8 (1.1–7.2); *p* value = 0.03	aOR 2.8 (1.1–7.2); *p* value = 0.03

Notes: aOR = adjusted odds ratio; 95% CI = 95% confidence interval. Genotype terms coded as shown in parentheses. All estimates reported from backward stepwise logistic regression outputs.

**Table 4:** Published minor allele frequencies (MAFs) by global populations vs. the current Jordanian cohort.

Variant	European MAF	African MAF	Asians MAF	Qatari MAF	Current Study MAF
rs1531380	32%	18%	13%	42%	48.6%
rs1531379	37%	25%	5%	42%	48.6%
rs1531378	32%	18%	13%	42%	48.6%
rs 6432860	33%	19%	13%	42%	48.6%
rs10198801	25%	5%	12%	24%	22.9%

**Table 5:** Characteristics of the primers used in the amplification of the region of interest for *SCN1A*.

Primer	Sequence (5′->3′)	Template Strand	Length	Position *	Tm	GC%	Self 5′ Complementarity	Self 3′ Complementarity
Forward	CTTTAGATAGCTCTTTCCTTCAGC	Plus	24	166040571–166040594	57.16	41.67	4.00	2.00
Reverse	CCACCCTAAACCATGTTCCC	Minus	20	166041782–166041763	58.15	55.00	4.00	0.00

* Position on accession number NC_000002.12. Tm: melting temperature as calculated by Primer-BLAST software program (version 2.5.0).

## Data Availability

The original contributions presented in this study are included in the article/Supplementary Material. Further inquiries can be directed to the corresponding authors.

## Supplementary Materials

The following supporting information can be downloaded at https://www.mdpi.com/article/10.3390/ph19050712/s1, Table S1. HWE. Exact Hardy–Weinberg equilibrium tests and MAF (QC and computation). Table S2. LOGREG. Multivariable Logistic Regression (dominant model; separate run per SNP; Multivariable model specification). Table S3. LD. Pairwise Linkage Disequilibrium (LD) among rs1531380, rs1531379, rs1531378, rs6432860, rs10198801. Figure S1. LD. Pairwise Linkage Disequilibrium (LD) among rs1531380, rs1531379, rs1531378, rs6432860, rs10198801. Figure S2. LD (triangle). Pairwise Linkage Disequilibrium (LD) among five *SCN1A* loci (rs1531380, rs1531379, rs1531378, rs6432860, rs10198801). Figure S3. LD (triangle, grayscale). Pairwise Linkage Disequilibrium among five *SCN1A* loci (rs1531380, rs1531379, rs1531378, rs6432860, rs10198801).
