# Supplementary material for: Genetic Polymorphisms in SCN1A Gene (rs6432860) and Pharmacoresistance to Antiepileptic Drugs Among Jordanian Patients with Epilepsy
Source: Pharmaceuticals (Basel). 2026 Apr 30;19(5):712. doi: 10.3390/ph19050712 (PMC13210710; doi:10.3390/ph19050712)
Supplement: Supplementary file 1 [file pharmaceuticals-19-00712-s001.zip › pharmaceuticals-4026567-supplementary.pdf]

Supplementary Materials

1	METHODS<br>**Table S1**. **HWE.** Exact Hardy–Weinberg equilibrium tests and MAF (QC and computation)
**2**	METHODS<br>**Table S2**. **LOGREG.** Multivariable Logistic Regression (dominant model; separate run per SNP; Multivariable model specification)
**3**	3.1. **Table S3. LD.** Pairwise Linkage Disequilibrium (LD) among rs1531380, rs1531379, rs1531378, rs6432860, rs10198801<br>3.2. **Figure S1. LD.** Pairwise Linkage Disequilibrium (LD) among rs1531380, rs1531379, rs1531378, rs6432860, rs10198801.
**4**	4.1. **Figure S2. LD (triangle).** Pairwise Linkage Disequilibrium (LD) among five SCN1A loci (rs1531380, rs1531379, rs1531378, rs6432860, rs10198801).<br>or<br>4.2. **Figure S3. LD (triangle, grayscale).** Pairwise Linkage Disequilibrium among five SCN1A loci (rs1531380, rs1531379, rs1531378, rs6432860, rs10198801).

1. S-HWE (QC and computation)

Exact Hardy–Weinberg equilibrium (HWE) test was evaluated for each SNP using the **exact test** (two-sided) on observed genotype counts, overall and stratified by response status. Results are reported as *P* values together with minor allele frequencies (MAF). Counts are those shown in **Table 2** of the main manuscript

Table S1. HWE. Hardy–Weinberg equilibrium and MAF.

SNP	Group	Genotypes (major hom / het / minor hom)	N	MAF	Exact HWE Tests *P value*
rs1531380	Overall	25 / 58 / 22	105	0.486	0.332
	Non-responders	5 / 27 / 11	43	0.430	0.118
	Responders	20 / 31 / 11	62	0.427	1.000
rs1531379	Overall	25 / 58 / 22	105	0.486	0.332
	Non-responders	5 / 27 / 11	43	0.430	0.118
	Responders	20 / 31 / 11	62	0.427	1.000
rs1531378	Overall	25 / 58 / 22	105	0.486	0.332
	Non-responders	5 / 27 / 11	43	0.430	0.118
	Responders	20 / 31 / 11	62	0.427	1.000
rs6432860	Overall	25 / 58 / 22	105	0.486	0.332
	Non-responders	5 / 27 / 11	43	0.430	0.118
	Responders	20 / 31 / 11	62	0.427	1.000
rs10198801	Overall	62 / 38 / 5	105	0.229	1.000
	Non-responders	29 / 13 / 1	43	0.174	1.000
	Responders	33 / 25 / 4	62	0.266	1.000

Abbreviations: hom: homozygote; het: heterozygote; MAF: Minor Allele Frequency. Counts mirror **Table 2**.

2. Supplementary Methods S-LOGREG (multivariable model specification)

- **Outcome:** Non-response to ASMs (binary; event = non-responder).
- **Genetic term:** Separate model per SNP under a **dominant coding** (minor-allele carriers vs. major-allele homozygotes), matching Table 2 contrasts.
- **Covariates: Age at interview** (years), **number of AEDs**, **carbamazepine use** (Yes/No).
- **Model selection: Backward stepwise logistic regression** (entry $\alpha = 0.05$; removal $\alpha = 0.10$), consistent with the main text Results.
- **Collinearity diagnostics: Variance inflation factors (VIF)** and **condition indices** were examined; no concerning multicollinearity was detected among covariates or with the genotype term.
- **Reporting:** Adjusted odds ratios (aOR) with 95% CI and two-sided *P value*. (Optional model fit indices—−2LL, Cox & Snell/Nagelkerke R^2, classification table—available on request.)
- **Haplotypes in modeling:** Due to **complete LD/concordance** among the four upstream SNPs and strong LD with rs10198801, haplotype terms were **not included together with single -SNP terms** to avoid redundancy; haplotype structure is summarized descriptively in **S-LD**

Table S2. LOGREG. Multivariable logistic regression (dominant model; separate run per SNP).

Covariate / Genetic term	Model: rs1531380 (TT+CT vs. CC)	Model: rs1531379 (AA+AG vs. GG)	Model: rs1531378 (AA+GA vs. GG)	Model: rs6432860 (AA+GA vs. GG)
Carbamazepine (Yes vs. No)	**aOR 3.3** (1.3–8.0); **P value = 0.009**	**3.3** (1.3–8.0); **P value = 0.009**	**3.3** (1.3–8.0); **P value = 0.009**	**3.3** (1.3–8.0); **P value = 0.009**
Number of AEDs	**0.17** (0.06–0.50); **P value = 0.002**	**0.17** (0.06–0.50); **P value = 0.002**	**0.17** (0.06–0.50); **P value = 0.002**	**0.17** (0.06–0.50); **P value = 0.002**
Age at interview (years)	**0.30** (0.10–0.61); **P value = 0.002**	**0.30** (0.10–0.61); **P value = 0.002**	**0.30** (0.10–0.61); **P value = 0.002**	**0.30** (0.10–0.61); **P value = 0.002**
Genotype (dominant)	**2.8** (1.1–7.2); **P value = 0.03**	**2.8** (1.1–7.2); **P value = 0.03**	**2.8** (1.1–7.2); **P value = 0.03**	**2.8** (1.1–7.2); **P value = 0.03**

3. Supplementary Table and Figure S-LD. Pairwise LD matrix (D′ and r^2) across the 5 SNPs

- **Display:** 5×5 matrix; **upper triangle = D′**, **lower triangle = r^2**; diagonal blank.
- **Key results (already in the main text):**
    - Upstream block (rs1531380–rs1531379–rs1531378–rs6432860): **D′ = 1** with complete concordance (*P* value< 0.001).
    - Upstream vs downstream (each upstream SNP with rs10198801): **D′ = 1**, **r ≈ –0.53** (thus **r^2 ≈ 0.28**), *P* value< 0.001.
- **Software:** Haploview v4.2, EM algorithm, default confidence intervals

Table S3. LD. Pairwise linkage disequilibrium (LD) among **rs1531380, rs1531379, rs1531378, rs6432860, rs10198801**

SNP_i	SNP_j	D'	r	r^2	P_value
rs1531380	rs1531379	1	1	1	<0.001
rs1531380	rs1531378	1	1	1	<0.001
rs1531380	rs6432860	1	1	1	<0.001
rs1531380	rs10198801	1	-0.53	0.281	<0.001
rs1531379	rs1531378	1	1	1	<0.001
rs1531379	rs6432860	1	1	1	<0.001
rs1531379	rs10198801	1	-0.53	0.281	<0.001
rs1531378	rs6432860	1	1	1	<0.001
rs1531378	rs10198801	1	-0.53	0.281	<0.001
rs6432860	rs10198801	1	-0.53	0.281	<0.001

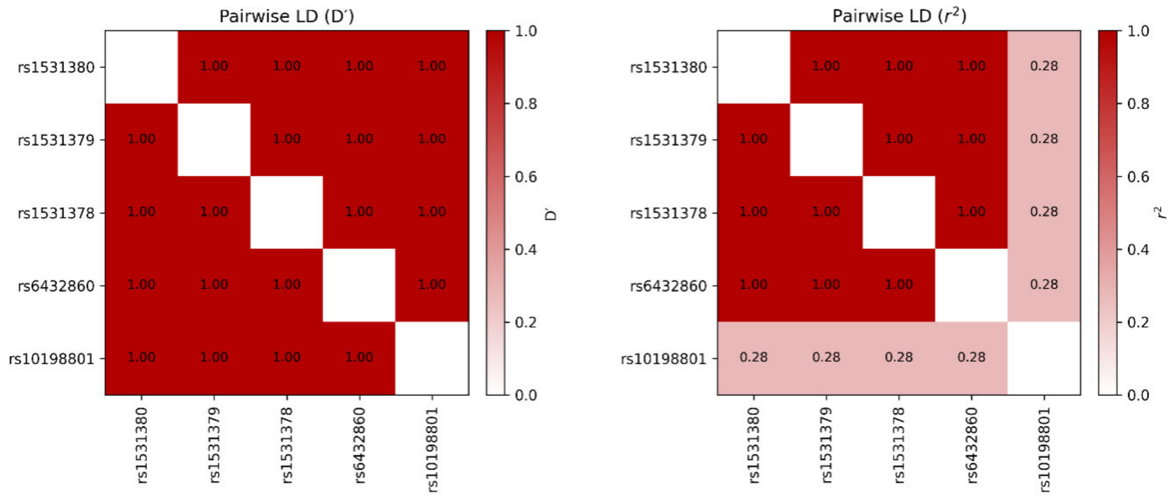

Figure S1. LD. Pairwise linkage disequilibrium (LD) among **rs1531380, rs1531379, rs1531378, rs6432860, rs10198801**. **(A)** Heatmap of **D′**; **(B) H**eatmap of **r^2**. The **four upstream SNPs** are in complete LD (**D′ = 1**) with perfect correlation (**r = 1; r^2 = 1**), while each upstream SNP and **rs10198801** show **D′ = 1** with negative correlation (**r ≈ –0.53; r^2 ≈ 0.28**); all comparisons ***P* value< 0.001**. Metrics and ordering match the main text

Alternatively;

4. Supplementary Figure S-LD (triangle). Pairwise linkage disequilibrium (LD) among five SCN1A loci (rs1531380, rs1531379, rs1531378, rs6432860, rs10198801).

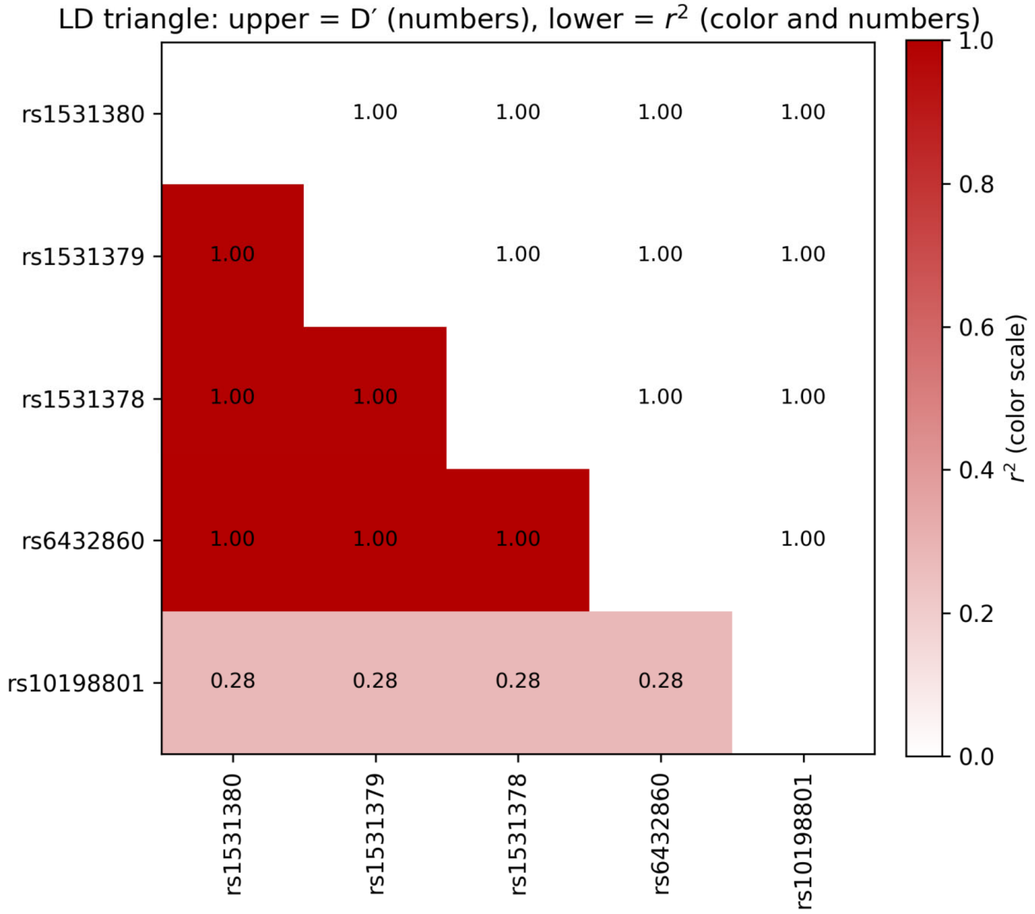

4.1. Figure S2. LD (triangle). Pairwise linkage disequilibrium (LD) among five SCN1A loci (rs1531380, rs1531379, rs1531378, rs6432860, rs10198801).

Legend: The **triangular matrix** displays **r²** as the color scale in the **lower triangle** (also printed numerically in each lower-triangle cell) and **D′** as numeric labels in the **upper triangle**. Axes are ordered **rs1531380** → **rs10198801** (left-to-right; top-to-bottom). As reported in the Results, the **four upstream variants** (rs1531380/rs1531379/rs1531378/rs6432860) are in **complete LD** (**D′ = 1**) with **perfect correlation** (**r = 1; r² = 1**), and each upstream variant with **rs10198801** shows **D′ = 1** with **negative correlation** (**r ≈ −0.53; r² ≈ 0.28**); all tests ***P* value< 0.001**. LD metrics and haplotypes were estimated using **Haploview v4.2 (EM algorithm)** on the genotypes described in the main text. See **Supplementary Table S-LD** for the numeric pairwise **D′/r/r²** values.

Or,

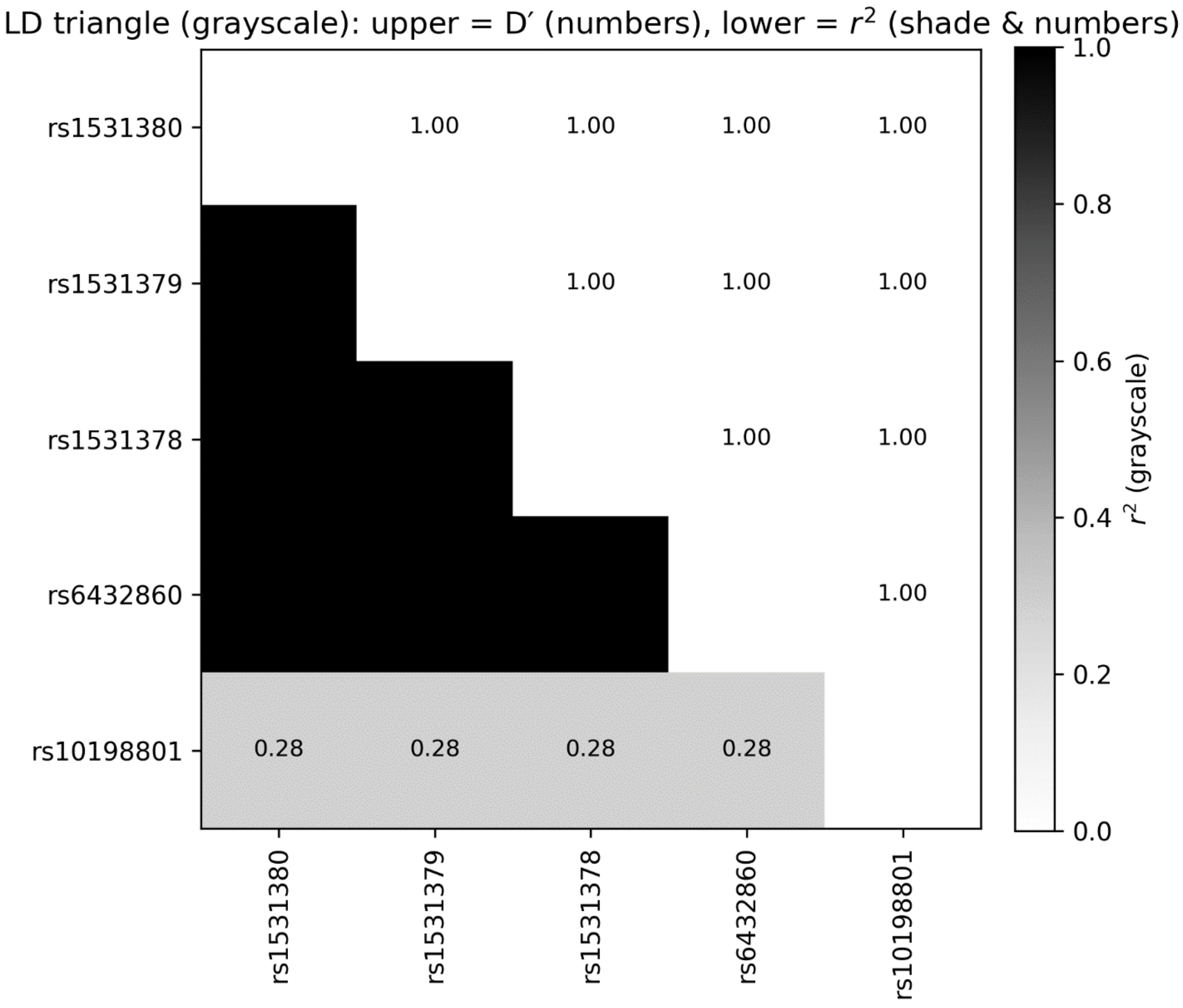

4.2. Figure S3. LD (triangle, grayscale). Pairwise linkage disequilibrium among five ***SCN1A*** loci (rs1531380, rs1531379, rs1531378, rs6432860, rs10198801).

Legend: The **lower triangle** displays $\mathbf{r^2}$ as grayscale shading (0 to 1; darker = higher LD) with numeric r^2 values overlaid; the **upper triangle** shows **D′** as numeric labels. Axis order is **rs1531380** → **rs10198801** (left-to-right; top-to-bottom). As detailed in the main text, the **four upstream variants** (rs1531380/rs1531379/rs1531378/rs6432860) are in **complete LD** (**D′ = 1**) with **perfect correlation** (**r = 1; r^2 = 1**), and each upstream variant with **rs10198801** shows **D′ = 1** with **negative correlation** (**r ≈ −0.53; r^2 ≈ 0.28**); all LD tests ***P value*** **< 0.001**. LD metrics/haplotypes were estimated in **Haploview v4.2** (EM algorithm) from the genotypes described in the main text; numeric pairwise values are provided in **Supplementary Table S-LD**
